# A New Methodology Based on Cell-Wall Hole Analysis for the Structure-Acoustic Absorption Correlation on Polyurethane Foams

**DOI:** 10.3390/polym14091807

**Published:** 2022-04-28

**Authors:** Beatriz Merillas, Fernando Villafañe, Miguel Ángel Rodríguez-Pérez

**Affiliations:** 1 Cellular Materials Laboratory (CellMat), Condensed Matter Physics Department, Faculty of Science, University of Valladolid, Campus Miguel Delibes, Paseo de Belén 7, 47011 Valladolid, Spain; marrod@fmc.uva.es; 2 GIR MIOMeT-IU Cinquima-Inorganic Chemistry, Faculty of Science, University of Valladolid, Campus Miguel Delibes, Paseo de Belén 7, 47011 Valladolid, Spain; fernando.villafane@uva.es; 3 BioEcoUVA Research Institute on Bioeconomy, University of Valladolid, 47011 Valladolid, Spain

**Keywords:** acoustic absorption, cellular structure analysis, cell wall holes, polyurethane foams

## Abstract

Polyurethane foams with a hybrid structure between closed cell and open cell were fabricated and fully characterized. Sound absorption measurements were carried out in order to assess their acoustic performance at different frequency ranges. The cellular structure of these systems was studied in detail by defining some novel structural parameters that characterize the cell wall openings such as the average surface of holes (*S_h_*), the number of holes (*h*), and the area percentage thereof (%HCW). Therefore, these parameters allow to analyze quantitatively the effect of different structural factors on the acoustic absorption performance. It has been found that the parameters under study have a remarkable influence on the normalized acoustic absorption coefficient at different frequency ranges. In particular, it has been demonstrated that increasing the surface of the holes and the percentage of holes in the cell walls allows increasing the acoustic absorption of these types of foams, a promising statement for developing highly efficient acoustic insulators. Additionally, we could determine that a suitable minimum value of hole surface to reach the highest sound dissipation for these samples exists.

## 1. Introduction

Currently, one major environmental problem that is causing an undesirable and negative impact on human health is noise pollution. The World Health Organization (WHO) states that one in five Europeans is regularly exposed to sound levels at night that could significantly damage health. Therefore, this concern has led to increasingly hardened regulations under the European Directive on the Assessment and Management of Environmental Noise [1]. For this reason, there is a huge need of understanding the key parameters for developing innovative and efficient sound insulating materials.

Porous materials are among the most commonly used as noise absorbers since the sound waves are allowed to penetrate and dissipate inside their complex structure. Their capacity to dissipate sound energy is mainly based the inertial loss promoted by the friction of air with the pore walls and thermal damping by thermal effects in the proximity of the solid walls [2,3,4]. Specifically, polyurethane (PU) foams have attracted significant attention as sound absorbers due to their versatility, cost effectiveness and high acoustic absorption efficiency. Owing to the great number of components (polyol, isocyanate, catalysts, surfactants, chain extenders, etc.) and parameters to be changed during the PU foam manufacturing, the modification of their formulations has been employed as a strategy to enhance their noise absorbance [5,6]. Most of the research is focused on flexible PU foams due to their excellent noise absorption capacities [7], which can be further enhanced by the addition of fillers [8,9,10].

Several studies have focused their efforts on determining the effect of different structural parameters on the noise absorption capacity of porous materials. Sedeq [11] evaluated the effect of different factors influencing the sound absorption of fibrous materials. A relationship between airflow resistance and noise absorption was found in which the latter increases when the airflow resistance is higher. Vašina et al. [12] investigated the sound absorption properties of porous materials, concluding that structure, thickness and air gap size are factors that have a strong influence. Yasunaga et al. [13] established a relationship between air flow and the effective fraction of open windows obtained by the cell opening mechanism in flexible polyurethane foams. In the latter work, cell windows were classified into fully open, partially open, pin holes and closed cells, concluding that a higher effective open window fraction leads to a higher air flow. Zhang et al. [14] also studied the effect of the open cell content and cell size on the acoustic properties for flexible PU foams, finding a clear relationship. Basirjafari [15] analyzed the effect of adding different CNT contents to flexible polyurethane foams on their morphological and acoustic properties. By evaluating the strut length to strut thickness ratio (L/t), a relation was found between the cell wall flexibility and the sound absorption. The optimum CNT amount was 0.05 wt.%, achieving the highest L/t ratio, which leads to a more effective damping mechanism.

**Figure 1:**
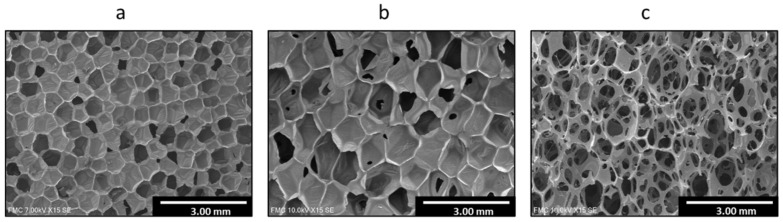
Typical cellular structures for closed cell rigid PU foam (**a**), hybrid rigid PU foam (**b**) and open cell PU foam (**c**).

While there is wide literature focused on the acoustic absorption of flexible PU foams [16,17,18,19,20,21], the studies on the acoustic behavior of rigid PU foams with a partially interconnected cellular structure is very limited. In fact, as far as the authors of this research know, there are no previous works for this type of material analyzing exhaustively and quantitatively the effect of the cellular structure parameters on the acoustic absorption. As previously indicated, most of the published works have evaluated the effect of different factors on the final acoustic properties. However, those which study the influence of the cellular structure present other changing features such as density, not allowing to understand the structural effects separately. Herein we study the acoustic behavior of polyurethane systems with significant stiffness of the polymeric matrix. These systems present an intermediate cellular structure architecture between the convectional closed-cell foams, where cells are polyhedra with solid faces (Figure 1a) and the typical open-cell structures of flexible PU foams (Figure 1c) in which most of the cell walls are lost and the foam is mainly composed of edges. In this research, hybrid cellular structures (Figure 1b), in which the degree of interconnection between cells is noticeably different, are considered. In these materials, the interconnection between cells is caused by holes in the cell walls. These hybrid cellular structures have been previously studied for ethylene butyl acrylate copolymer foams [22] and polyolefin foams [23], both of them flexible materials but not for rigid PU foams.

In order to carry out a systematic study, a new approach to characterizing some key parameters of the cellular structure of these materials is presented in this paper. Herein we analyzed the effect of the holes present in the cell walls and their main characteristics on the acoustic performance of rigid PU foams. Density, open cell content and cell size are kept almost constant and by variations in the catalyst concentration other structural parameters were modified to evaluate its influence on the acoustic performance.

## 2. Materials and Methods

### 2.1. Materials

The formulations of this work are bi-component rigid polyurethane foams. The isocyanate component is a polymeric diphenylmethane diisocyanate (pMDI) (IsoPMDI 92140) (density = 1.23 g/cm^3^) provided by BASF Poliuretanos Iberia S.A. (Barcelona, Spain). The polyol component includes a mixture of two polyols (VORANOL™ CP 450 (50% by weight) and VORANOL™ CP 1055 (50% by weight)) obtained from DOW (Midland, MI, USA). The mixture of polyols is considered as 100 parts by weight and the rest of the components are expressed in relation to this value. Distilled water was used as a chemical blowing agent (4.8 ppw). Additionally, an additive based on carbon black (TEGOCOLOR^®^ BLACK HI) (0.3 ppw), TEGOSTAB^®^ B 8522 (non-hydrolysable poly-ether-polydimethyl-siloxane) (1 ppw), employed as surfactant, and two amine-based catalysts (TEGOAMIN^®^ DMCHA and TEGOAMIN^®^ PMDETA) were supplied by Evonik (Essen, Germany). The different foams differed on the catalyst amount as follows: PU1 (DMCHA 0.5 ppw, PMDTA 0.5 ppw), PU2 (DMCHA 0.5 ppw, PMDTA 0.75 ppw), PU3 (DMCHA 0.75 ppw, PMDTA 0.75 ppw), PU4 (DMCHA 0.75 ppw, PMDTA 0.5 ppw), PU5 (DMCHA 0.5 ppw, PMDTA 0.3 ppw). The proportion between isocyanate and polyol blend was 1.7:1.0.

### 2.2. Fabrication Procedure

A mixture of polyols and additives was homogenized by stirring at 250 rpm for ten minutes with a low-shear fluid mixing machine (EUROSTAR Power control-visc P1, IKA, Staufen, Germany) provided with a 50 mm diameter Lenart disc. The polyurethane foams were prepared by mixing the isocyanate and polyol blend (isocyanate-polyol ratio of 1.7:1.0) at 2000 rpm for ten seconds in a plastic bucket The mixture was poured into a paperboard box and was allowed to react by free-rising. After 48 h of ageing at room temperature, samples were saw-cut, obtaining cylinders with 30 mm diameter and 10 mm thickness.

### 2.3. Foams Characterization

#### 2.3.1. Density

Geometrical density (ρ) was measured as described by ASTM D1622/D1622M 14 [24]. One cylindrical sample of 30 mm × 10 mm (diameter × height) was measured for each formulation. Relative density (ρ_r_) (the ratio between density of the foam and density of solid PU) was calculated by using 1160 kg/m^3^ as the solid density of the polyurethane matrix.

#### 2.3.2. Open Cell Content

The open cell content (OC%) was determined through nitrogen pycnometry with an Accupyc II 1340 from Micromeritics, Georgia, USA, according to ASTM D6226-10 [25]. A cylindrical sample of each formulation was employed to carry out this measurement.

#### 2.3.3. Acoustic Properties

The sound absorption properties were obtained by using a two-microphone impedance measurement tube from Bruel & Kjaer, Nærum, Denmark, type 4206, UA-1630, with a frequency range of 500–6500 Hz at an exposure time of 4.3 s. The measurements were performed according to the standard procedure detailed in ASTM E1050-10 [26]. The acoustic absorption coefficient is defined as the ratio of the absorbed sound energy at a specific frequency range by the polyurethane foam (I_incident_–I_reflected_) to the incident energy (I_incident_) on the sample surface. The absorption coefficient was calculated as the average value of six measurements (three for each of the cylinder bases) to obtain consistent results. The selected cylinder (30 mm diameter × 10 mm height) for the acoustic measurements was the same as that used for the density and open cell calculations to ensure the accuracy of the data. The incident wave was parallel to the foam rise direction.

#### 2.3.4. Cellular Structure Analysis

The cellular structure micrographs were acquired by using scanning electron microscopy (SEM) with a FlexSEM 1000 Hitachi (Tokio, Japan) microscope. The foams were saw-cut and the surface was examined by SEM after vacuum coating with a gold monolayer. The evaluated plane is the growth plane of the foams (z). The SEM images were analyzed with a software based on Image J/FIJI [27] obtaining the main characteristics of the cellular structure: Average cell size (Φ_3*D*_), number of cells (*n*) in the analyzed total cell area (*S_t_*), average surface of the cells (*S_p_*), number of holes (*h*) in the analyzed area and the average surface of the holes (*S_h_*), i.e., the average area of the holes detected in the analyzed area for each sample.

**Figure 2:**
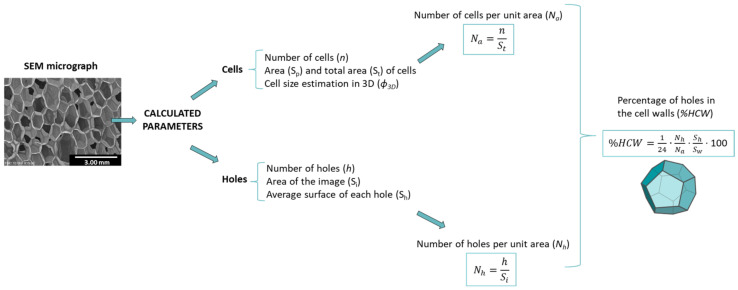
Cellular structure characterization methodology.

Figure 2 shows the different parameters calculated from the previous ones.

The samples selected for the structural analysis were the same as used for the acoustic absorption, density and open cell measurements. For each sample, a number of cells (*n*) greater than 100 was analyzed. The number of cells per unit area (*N_a_*) and the number of holes per unit area (*N_h_*) were calculated (see Figure 2). The analysis of the holes was carried out by employing scanning electron micrographs with a higher magnification than that used for the analysis of the cells. The same procedure was used selecting a number of holes (*h*) higher than 30 and estimating its average surface (*S_h_*). Through the calculation of the cell size, assuming a pentagonal dodecahedron geometry [28], the average surface of each cell wall can be calculated (*S_w_*). To obtain this value, first Equation (1) relates the volume of a sphere and the volume of a pentagonal dodecahedron to extract the pentagonal side (*L*)-cell size (Φ_3*D*_) relationship (Equation (2)).

(1) $$V=\frac{4}{3}\pi (\frac{\Phi_{3D}}{2}{)}^{3}=\frac{1}{4}\;\left(15+7\sqrt{5}\right)L^{3}$$

(2) $$L\approx 0.4088\;\Phi_{3D}$$

Then, with the expression of the dodecahedron area (Equation (3)), the surface area of each wall (*S_w_*) can be estimated:

(3) $$A=20.68\;L^{2}\approx 3.45\;{\Phi_{3D}}^{2}$$

(4) $$S_{w}=0.288\;{\Phi_{3D}}^{2}$$

Finally, taking into account that *S_w_* can be obtained from the average cell size, the percentage of area occupied by the holes in the cell walls (%*HCW*) was calculated by using Equation (5):

(5) $$\%HCW=\frac{1}{24}\frac{N_{h}}{N_{a}}\frac{S_{h}}{S_{w}}\cdot 100$$

The factor 1/24 takes into account that each cell is composed of 12 walls that are shared between two cells. The relationship *N_h_*/*N_a_* gives the number of holes per cell (NHP).

## 3. Results and Discussion

### 3.1. Foam Properties

The main characteristics of the PU foams are listed in Table 1. There are several works where a relationship between the porous material density and the acoustic absorption coefficient has been found [11,29,30]. Therefore, in this work, the selected samples present similar geometrical and relative density values that are around 27 kg/m^3^ and 0.023, respectively. The effect of the open cell content on sound absorption for polyurethane foams was studied by Park et al. [31] by theoretically modelling, supported by experimental results, the sound absorbing performance. Since the open cell content is a factor that influences the acoustic performance, and the aim of this work is to study the effect of other microstructural parameters, foams were produced in a narrow open cell content ranging between 80 and 90% (Table 1). In this way, it is expected that this structural parameter does not make the difference on the acoustic absorption behavior. The strategy used for keeping these parameters almost constant (density, open cell content and cell size) while producing variations on the cellular structure and sound absorption performance is based on the modification of the PU formulations. The study of the reaction kinetics is a crucial factor for controlling the final density and cellular structure of these materials. Modifications on the amounts of gelling and blowing catalysts produce significant changes on the polymerization and foaming reactions, respectively, and thus on the cellular structure. Additionally, the surfactants added to the formulations can act as stabilizers of the foam structure, as cell openers and as reducers of the surface tension between PU and air [32,33].

### 3.2. Cellular Structure Characterization

Some features of the cellular structure, such as cell size and distribution or cell holes, may cause a variation on the final sound absorption capacity. The structural parameters are described and analyzed in this section.

**Figure 3:**
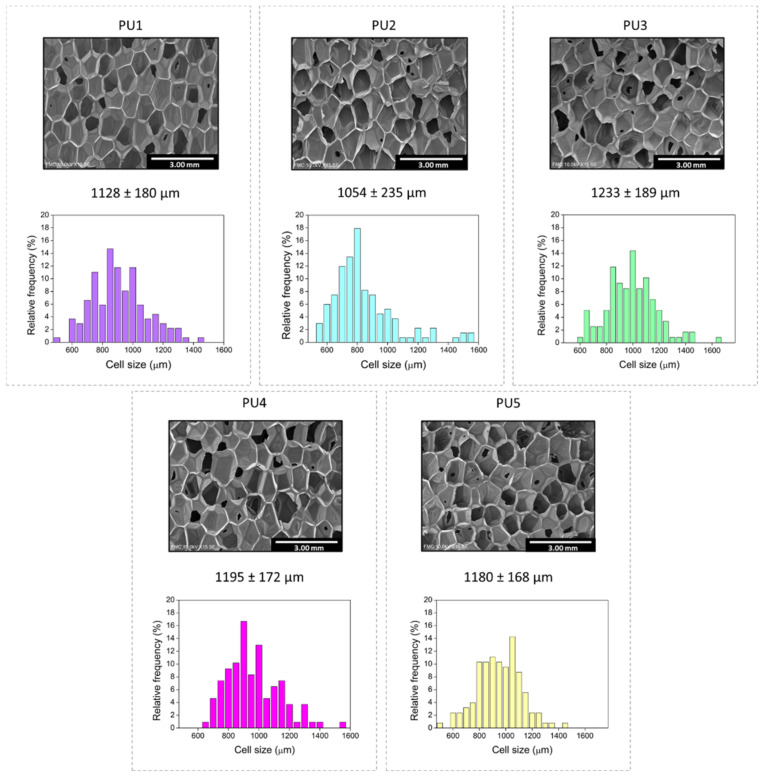
Cell size distribution and scanning electron micrographs of all the samples.

Cell size distributions and cellular structure micrographs are displayed in Figure 3. Pore sizes range from 1000 to 1250 microns with narrow pore size distributions, accounting for the cell homogeneity of the fabricated foams. The foams PU1, PU4 and PU5 show the narrower pore size distributions, whereas the samples PU2 and PU3 cover a slightly greater range.

**Figure 4:**
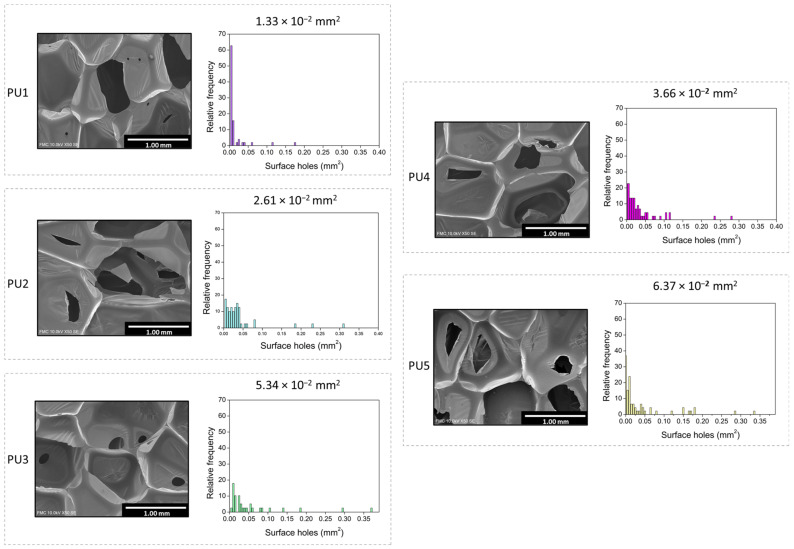
Surface hole distribution for each sample and micrographs for representative holes in the cell walls.

An exhaustive analysis of the holes in the pore walls was carried out. A similar image area was selected for each sample and the number, size, and surface of holes were estimated (see Figure 2). The hole area distributions are plotted in Figure 4, as well as the corresponding micrographs showing the representative type of holes for each foam. Additionally, the numerical values can be found in Table 2. The PU1 sample displays the holes with the smallest size, with 1.33·10^−2^ mm^2^ as average value, and the narrowest distribution. Samples PU2 and PU4 present bigger hole sizes (2.61·10^−2^ and 3.66·10^−2^ mm^2^ respectively), followed by PU3 (with 5.34·10^−2^ mm^2^). The system with the largest average hole size is PU5 (6.37·10^−2^ mm^2^), showing a distribution which is more heterogeneous than those for the rest of the formulations.

The number of holes per unit area (*N_h_*) was calculated as described in Figure 2. An inverse relationship with the average surface hole value is obtained. When holes present a smaller area, the number of holes per unit area is higher, obtaining the maximum value of 0.44 mm^−2^ for PU1. Nevertheless, according to the open cell values, in the case of holes with a larger area, *N_h_* decreases, reaching 0.34 mm^−2^ for PU5. The parameter defining the percentage of holes per cell wall (% HCW) covers a range between 0.045 for PU1 and 0.167 for PU5, following the same trend as the surface of holes as a result of its influence on this parameter.

### 3.3. Acoustic Properties

**Figure 5:**
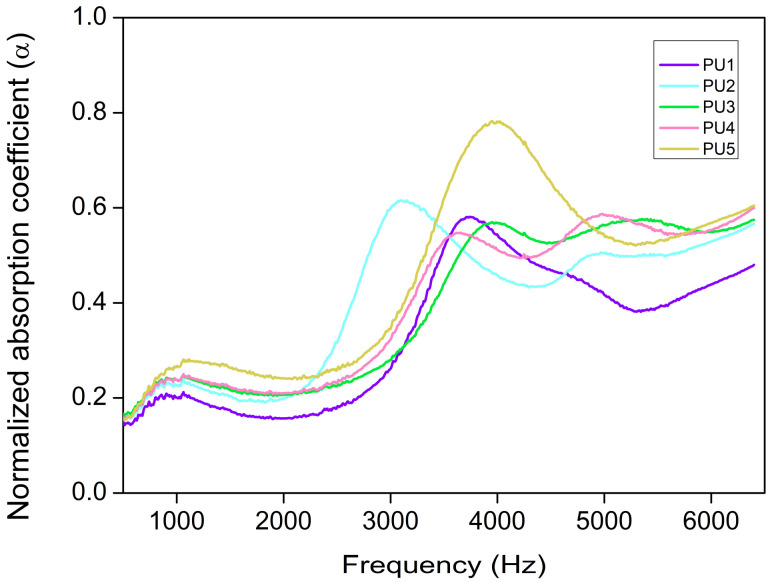
Acoustic absorption curves in the frequency range of 500–6500 Hz.

The acoustic measurements were carried out throughout a frequency range from 500 to 6500 Hz, and the acoustic performance was evaluated by the sound absorption coefficient. The acoustic absorption coefficient along the selected frequencies is represented in Figure 5. PU5 shows a notably higher acoustic absorption than the other samples throughout the whole frequency range, having two maximum peaks: One at low (ca. 1100 Hz) and one at high frequencies (ca. 4000 Hz). However, PU2, PU3 and PU4 systems show a different absorption curve, with three maximum peaks instead of two. This third peak appears at frequencies above 5000 Hz. An intermediate behavior is found for PU1, it being the formulation with the lowest absorption coefficient for almost all the frequencies.

The normalized acoustic coefficient was calculated for each system by the area integration of the average absorption curve through the following equation [23]:

(6) $$\alpha =\frac{\int_{f_{1}}^{f_{2}}\alpha \left(f\right)df}{f_{2}-f_{1}}$$

**Figure 6:**
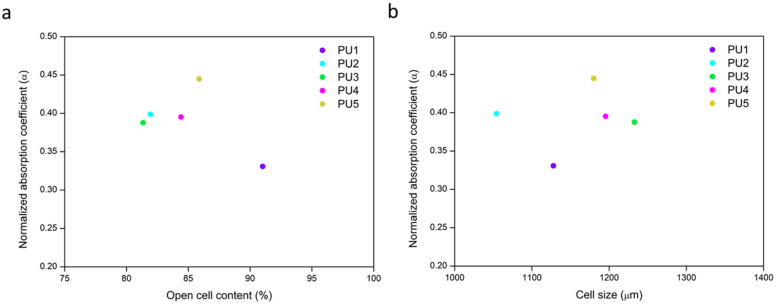
Relationship between the normalized absorption coefficient (*α*) between 500 and 6500 Hz and the open cell content (**a**); cell size (**b**) for all the samples.

Aiming to find a possible relationship between the normalized absorption coefficient (*α*) (500–6500 Hz) and the open cell content (Figure 6a) or cell size (Figure 6b), these parameters were plotted.

As shown in Figure 6a the variation of the acoustic properties of these samples is not related to the open cell content since different absorption coefficient values are found for almost similar open cell contents (samples PU2, PU3, PU4 and PU5). In fact, despite the general assumption that the higher the open cell content, the greater the acoustic performance [23], PU1 presents the highest open cell content, 91.02%, but its normalized absorption coefficient is the lowest one. Additionally, cell size is considered to be a factor determining the final acoustic absorption in porous materials since smaller cells lead to an increase in the airflow resistivity and, therefore, it will be harder for the sound wave to penetrate [34]. Nevertheless, due to the narrow interval of cell sizes that these samples show, there is not a clear relationship between the average cell size and the normalized absorption coefficient (Figure 6b). Therefore, the acoustical differences between samples should be connected to the other structural parameters described in the previous section (*S_h_*, *N_h_* and %*HCW*).

Table 3 gathers the normalized absorption coefficient (*α*) for each sample. Different ranges covering 2000 Hz were selected to determine possible variations on the absorption trend depending on the frequency range. As already observed in Figure 5, PU1 is the sample with the lowest absorption coefficient for all the frequencies. The foams with labels PU2, PU3 and PU4 show similar absorption coefficients. As has been seen before, the sample PU5 presents the highest values for all the ranges, reaching an average value of 0.44 for the complete range of frequencies. These numerical values follow the same trend as the surface holes of Figure 4, suggesting a direct relationship between the acoustic absorption properties and the size of the holes on the cell walls.

A deeper analysis of the influence of the structural parameters and the acoustic performance of these foams can be found in the following section.

### 3.4. Relationship between Cellular Structure and Sound Absorption

**Figure 7:**
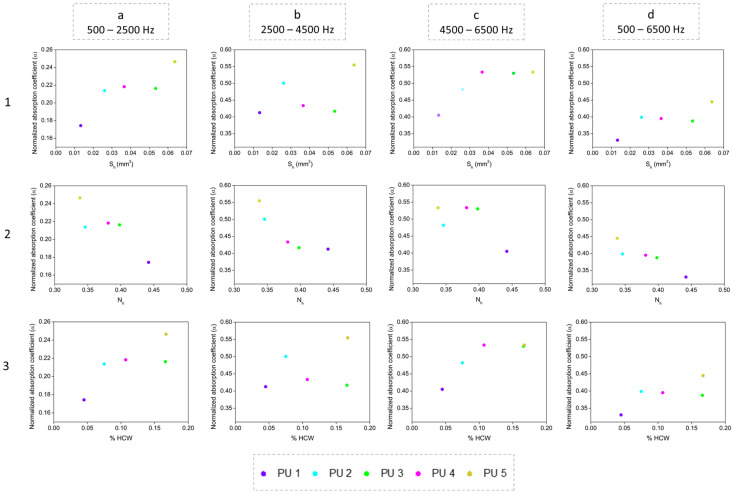
Relationship between surface hole *S_h_* (first row), number of holes *N_h_* (second row) and percentage of holes’ area on the cell walls %HCW (third row) with the normalized absorption coefficient at 500–2500 Hz (column (**a**)), 2500–4500 Hz (column (**b**)), 4500–6500 Hz (column (**c**)) and the complete range 500–6500 Hz (column (**d**)).

The results discussed above indicate that density, open cell content and cell size are not the critical factors controlling the differences in the sound absorption capacity of the samples under study since there is a slight variation in their numerical values. Thus, there must be other structural parameters contributing to govern the sound properties of the polyurethane foams. Aiming to look for them, other parameters of the cellular structure were analyzed. Thus, a noticeable relationship between the acoustic behavior and both the number and the surface of holes which are present on the cell walls was found. Figure 7 shows the relationship between the average surface of holes *S_h_* (first row), the number of holes per unit area *N_h_* (second row) and the percentage of holes’ area on the cell walls %*HCW* (third row) with the normalized absorption coefficient for every range of frequencies selected. The obtained results indicate that the higher the surface of holes (*S_h_*), the higher the acoustic absorption coefficient (first row). This trend was observed for all the frequency ranges under study (columns a–c).

These results confirm that the sound absorption capacity of porous materials mainly depends on the pore interconnection. It is well known that the efficiency of sound absorption is based on a low sound reflection and a high sound dissipation. The acoustic waves lead to the vibration of the cell walls through stretching and bending; thus, cell walls and the air inside pores contribute to dissipating the sound energy by vibration damping [8]. The normalized acoustic coefficient increase when pores have a higher surface may be due to the requirement that a minimum specific hole surface reach an efficient sound dissipation.

For higher frequencies (4500–6500 Hz) the maximum absorption capacity is reached at a *S_h_* value of 0.04 mm^2^. However, for lower frequencies (500–2500 Hz and 2500–4500 Hz), this limiting value of *S_h_* was not found in this study since the acoustic coefficient continued increasing with the surface area of holes. Regarding the general trend for the whole frequency range (d column), the acoustic coefficient is similar when *S_h_* varies between 0.025 and 0.055 mm^2^, showing a plateau region, and it has a clear improvement for surface holes that reach a value higher than 0.060 mm^2^, as is the case of PU5.

Regarding the number of holes *N_h_* (second row), the same tendency is shown for all the frequency ranges; that is, more holes reduce the sound absorption efficiency. This behavior might be explained by means of the sample tortuosity. For a fixed value of surface of holes, when the number of holes is lower, tortuosity increases, leading to a high air flow resistance by means of friction of viscosity through the vibration of air [10,11]. Thus, an increment on the path tortuosity implies a higher sound coefficient since the acoustic wave has more difficulty concerning passing through the porous sample.

Therefore, the *N_h_* parameter has an inverse relationship with the sound coefficient, whereas the surface of holes *S_h_* presented a direct one since the sound absorption capacity is higher as this parameter increases.

**Figure 8:**
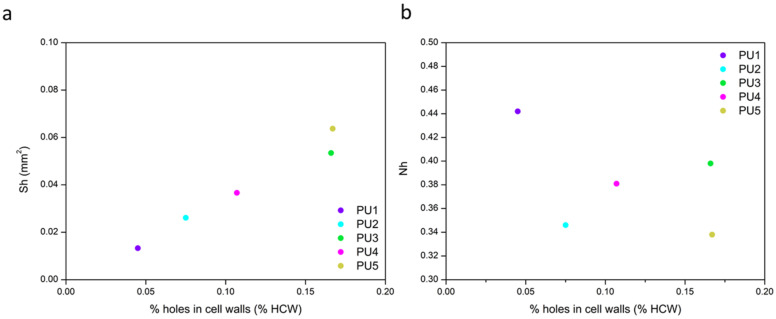
Relationship between the average surface of holes (**a**) and the number of holes (**b**) with the percentage of holes in the cell walls.

Finally, the third row of Figure 7 shows the percentage of holes’ area per cell wall (%*HCW*) that takes into account the number and surface of holes (*N_h_* and *S_h_*, respectively), as well as the number of cells (*N_a_*) and the average area of cell walls (*S_w_*), as shown in Equation (5). This structural parameter follows the same trend as the surface of holes, confirming that the percentage of holes’ area per wall has to be increased in order to enhance the acoustic behavior of rigid polyurethane foams. The relevance of *N_h_* and *S_h_* contributions on the final value of %*HCW* can be analysed by plotting each of these two parameters versus the %*HCW*, as shown in Figure 8.

Only the average surface of holes shows a direct correlation with the final percentage of holes’ area present in the cell walls, even though both parameters multiply in Equation (5). However, the values of the number of holes per unit area (*N_h_*) for all the samples under study differ in a narrow range; hence, its contribution to the final result has a lower impact.

Consequently, when considering a specific percentage of holes’ area in the cell walls, the results obtained indicate that a more significant improvement on the sound absorption coefficient should be obtained when there are fewer holes with a higher surface, rather than smaller holes in a higher number.

## 4. Conclusions

A new methodology aiming to characterize hybrid foam structures and relate the key parameters connecting the holes in the cell walls with the acoustic absorption coefficient (at different frequency ranges) was successfully developed.

Several rigid PU foams with cell size values in a narrow range (1000–1250 µm), having similar densities (around 27 kg/m^3^), and similar open cell contents (80–91%) were fabricated. These systems present an intermediate cellular structure between the conventional rigid closed cell PU foams and flexible open cell PU foams. Thus, their main peculiarities, apart from the high stiffness of the polymer matrix, are the holes present in the cell walls that are the cause of their sound absorption capacity.

An exhaustive structural characterization was carried out by defining new parameters in order to understand the relationship between the cellular structure and the acoustic absorption properties. The novel parameters introduced in this work are the area of the holes (*S_h_*), the number of holes per unit area (*N_h_*) and the percentage of cell wall area occupied by these holes (%*HCW*). Since tortuosity will change depending on the number and surface of holes, an equilibrium between these two factors should be reached for an optimum sound absorption. The obtained results show that a noticeable connection exists between the area of the holes (*S_h_*) and the final acoustic performance, obtaining a minimum surface hole of 0.04 mm^2^ for reaching the highest absorption coefficient between 4500 and 6500 Hz. In addition, the percentage of cell wall area (%*HCW*) also shows a direct correlation with the absorption coefficient, being larger when these parameters increase. Therefore, these results indicate that a small number of large size holes in the cell walls is the ideal structure to maximize the acoustic absorption of these rigid PU foams.

## Figures and Tables

**Table 1:** Properties of the foams.

Sample	Density (ρ) (kg/m^3^)	Relative Density (ρ_r_)	Open Cell Content (%)
PU1	27.74	0.024	91.02
PU2	27.58	0.024	81.94
PU3	27.37	0.024	81.34
PU4	27.14	0.023	84.41
PU5	27.05	0.023	85.88

**Table 2:** Structural parameters of the foams under study.

Sample	Surface Holes (*S_h_*) (mm^2^)	*N_h_* (mm^−2^)	%HCW
PU1	1.33 × 10^−2^	0.44	0.045
PU2	2.61 × 10^−2^	0.35	0.075
PU3	5.34 × 10^−2^	0.40	0.166
PU4	3.66 × 10^−2^	0.38	0.107
PU5	6.37 × 10^−2^	0.34	0.167

**Table 3:** Normalized absorption coefficient at different frequency ranges.

Sample	*α*_500–2500 Hz_	*α*_2500–4500 Hz_	*α*_4500–6500 Hz_	*α*_500–6500 Hz_
PU1	0.1743	0.4127	0.4051	0.3307
PU2	0.2138	0.5006	0.4819	0.3988
PU3	0.2163	0.4169	0.5301	0.3877
PU4	0.2183	0.4336	0.5336	0.3952
PU5	0.2465	0.5548	0.5335	0.4449

## Data Availability

Not applicable.
